# Self-Efficacy in the Prediction of GPA and Academic Computer Use in Undergraduate Translation Students at a Saudi University

**DOI:** 10.3389/fpsyg.2022.865581

**Published:** 2022-06-06

**Authors:** Abbas Brashi

**Affiliations:** English Language Department, College of Social Sciences, Umm Al-Qura University, Mecca, Saudi Arabia

**Keywords:** translation studies, COVID-19, online learning, general self-efficacy scale, computer self-efficacy scale, self-directed learning, translation self-efficacy framework

## Abstract

Since the beginning of the COVID-19 pandemic, academic institutions have faced the challenge of understanding the social-psychological features that produce better academic performance among translation students in an online learning environment. Although self-efficacy is widely studied in a variety of teaching and learning models, few studies have examined self-efficacy in regard to translation students. This empirical study aims to examine the roles of general self-efficacy and computer self-efficacy in terms of the academic achievement and computer use of translation students during the COVID-19 pandemic. The participants included 83 undergraduate translation students at the English Language Department of Umm Al-Qura University, Saudi Arabia. They completed the General Self-Efficacy Scale and Computer Self-Efficacy Scale questionnaires. The results found self-efficacy scores were a significant predictor of overall GPA scores, highly predictive of computer self-efficacy, and also predicted an increased typing frequency and computer usage. This study suggests that translation students with greater levels of self-efficacy will be more likely to possess social-psychological features that align with the independent, self-directed nature of online learning environments.

## Introduction

Bandura (1977) was the first to propose self-efficacy as a psychological construct which can be described as “a belief about one’s own capability to organize and complete a course of action required to accomplish a specific task” (Eggen and Kauchak, 2007: 310). Bandura (1977) states that self-efficacy is concerned with self-judgments about what one can do with their skills. This conceptualization of self-efficacy has two components. The first is efficacy expectations, focusing on one’s belief in their individual capacity to affect behavior. The second is outcome expectations, entailing how an individual believes that their behavior will lead to specific outcomes (Albion, 1999).

Based on the source of self-judgment, individuals can have negative or positive perceptions about a behavior before it is conducted, potentially affecting whether or not they take part in the action (Bandura, 1986; Albion, 2001). Factors affecting these ideas and perceptions are based on their psychological state, verbal persuasion capabilities, past experiences, and behavior modeling (Bandura, 1986). When an individual performs well on one task, they are more likely to believe they will perform well on a similar task (Eggen and Kauchak, 2007). Self-efficacy is also increased by observing others successfully performing similar behaviors. Verbal persuasion can encourage people to complete tasks, while different psychological states, such as stress, anxiety, hunger, and fatigue for example, can make them believe they will not be successful in such a task (Scholz et al., 2002).

In terms of motivation and academic goals, self-efficacy appears to be a significant predictor of the amount and length of time an individual dedicates to completing a task. Self-efficacy also impacts an individual’s goals and the degree to which they can persevere through challenges (Khorrami-Arani, 2001). It is therefore associated with motivation and academic success, as individuals with this trait are often quicker to set goals for themselves and work through challenges without quitting prematurely. Additionally, when individuals with high self-efficacy fail at a task, they tend to re-assess the situation and think about how to recover quickly, seeking new strategies to achieve their goal (Scholz et al., 2002).

An individual can have high self-efficacy in one domain, such as golfing, while simultaneously possessing low self-efficacy in others, such as academic studies. Self-efficacy is therefore not a global marker of efficacy in all domains, but rather domain specific. As such, this trait has been studied regarding its relationship with a wide range of behavioral areas, such as medicine, psychology, business, and education.

Educational structures and platforms in countries all over the world have been drastically altered following the recent COVID-19 global public health emergency over the course of 2020 and 2021. On March 11, 2020, the World Health Organization officially stated that COVID-19 was a pandemic, spreading uncontrollably both within and between national borders. In Middle Eastern countries, many nations implemented a full lockdown, while others imposed only partial lockdowns. Educational sectors were among those most impacted by this development, facing strict lockdowns that effectively shifted physical learning environments toward online platforms.

Almahasees and Qassem (2021) argue that when it comes to translation studies in particular, the need to understand the material on a practical level requires a far greater degree of direct contact with instructors. They assert that “It is a *de facto* that teaching translation courses involve face to face communications, and the switch to online mode makes it necessary to scrutinize the faculty’ readiness to teach online and their perceptions of teaching translation courses regarding limitations, challenges, teaching, and assessment strategies” (Almahasees and Qassem, 2021, p. 2). They also argue that student-centered rather than teacher-centered instruction methods benefit from recent technological innovations. Specifically, student centered instruction aligns with online learning because the internet and technological tools to transfer, share, and extend knowledge help to promote a higher quality of learning.

This study seeks to analyze the association between self-efficacy on the one hand and technology and academic performance among undergraduate translation students on the other. It does this by examining the learning experiences of 83 undergraduate translation students at a Saudi Arabian University in the wake of the COVID-19 pandemic. The following sections will present a review of the literature, aims of the study, methodology, results, and a combined discussion and conclusion respectively.

## Literature Review

### The Impact of Distance Learning on Translation Studies

The COVID-19 pandemic has led to the widespread closure of educational institutions across the world. These circumstances have brought considerable attention concerning the importance of distance learning *via* online platforms. Almahasees and Qassem (2021) examined translation instructors’ perceptions of teaching online translation courses during the pandemic. They used an empirical model with a questionnaire regarding teaching strategies and associated challenges in determining students’ performance. The study was facilitated with the use of Zoom, Microsoft Teams, and WhatsApp (Almahasees and Qassem, 2021). They found that participants reported online education as being less efficacious than face to face learning. Additionally, students appear to have faced difficulties within the online learning environment and trouble adapting to this style of communication. They also reported problems concerning personal motivation and a lack of internet connections. As such, it was concluded that online education is not a sufficient replacement for face to face learning and that educators should attempt to combine both face to face learning and online education to create a more rigorous learning environment (Almahasees and Qassem, 2021).

In Arab countries, the use of online learning platforms is dependent upon the available infrastructure. The Levant countries, such as Jordan, Lebanon, Syria, and Palestine, and the Arab Gulf countries have quickly transitioned to online learning environments (Almahasees and Qassem, 2021). Jordan for example has been implementing an online learning infrastructure since 2002 *via* collaboration between the Planning and Information Technology ministries and the Ministry of Education. Over a decade ago, Saadeh and Al-Karimi (2009) found that students at the University of Jordan considered online learning an adequate replacement for face to face instruction. The COVID-19 pandemic has thus re-ignited the need to better understand which type of learning environments and technologies improve the quality of online learning platforms in Middle Eastern educational institutions.

Al-Jarf (2020) examined college students in Saudi Arabia during the COVID-19 pandemic, finding that 55% of students were not satisfied with online learning because this type of communication made it more difficult to understand the lecture material. The author also found students experienced an, “absence of goals, low self-efficacy, low student engagement and motivation, and a negative role” (Al-Jarf, 2020, p. 37). Research regarding the affects COVID-19 pandemic in this context has produced few studies, even though learning behaviors and perceptions may greatly impact academic success when using online platforms. Al-Jarf (2020) found that students and teachers use many online applications, such as Zoom, Google Classroom, Google Meet, WebEx, Microsoft Teams, and educational platforms such as Coursera and Blackboard. Yet while some students find them beneficial and efficacious, others feel that such platforms are ineffective and frustrating (Al-Jarf, 2020).

Al-Jarf (2020) found that 45% of students in a Saudi Arabian sample reported dissatisfaction with online distance learning, especially among students and teachers familiar with online learning and the use of Blackboard and web conferencing services before the beginning of the pandemic. As much as 55% of participants reported online distance learning to be a frustrating experience, preferring face to face instruction (Al-Jarf, 2020).

Research from Alwazna (2021), on the other hand, does not appear to support this. In this study, a questionnaire including both closed-ended and open-ended questions was used to gain a better understanding of sixty translation teachers’ online teaching experiences during the pandemic. Although many teachers suggested they had encountered problems at some point, 40% evaluated their experience as enjoyable and none claimed it was not good. As such, more research is arguably needed to examine how students in the Arab world use their psychological and behavioral faculties to perform better on tasks associated with online learning.

### The Possibility for a Differential Effect

Kalyanasundaram and Madhavi (2020) examined students’ perceptions of their online courses, finding most were optimistic about online learning during the pandemic (Almahasees and Qassem, 2021). They also found that online learning promoted a reliance on critical thinking and self-learning, unlike traditional teacher-centered learning (Almahasees and Qassem, 2021). Online learning strategies may therefore require a degree of positive self-perception concerning one’s own learning behaviors, processes, and self-knowledge.

Rojo-López and Naranjo (2021) examined the relationship between the emotional impact of contextually relevant source texts (STs) and behavior among translation students. The students completed translations on two English STs with varying evaluative attitudes about the COVID-19 pandemic, including pessimistic and optimistic framing. They found that optimistic versus pessimistic attitudes predicted translation strategies, levels of affect, and anxiety levels immediately after the exercises. Students for whom the COVID-19 pandemic was framed more optimistically showed a lower emotional attenuation in their translations, implying that pessimistic participants were more likely to alter the texts by “intensifying or attenuating the ST emotional content” (Rojo-López and Naranjo, 2021, p. 37).

This supports findings suggesting when one has higher attentional demands, task performance is affected to a greater extent by anxiety-driven cognitive processing (Alves et al., 2010). These findings also support previous research suggesting individuals using evaluative language are more likely to be affected by the contextual frames present during a crisis (Chiluwa and Ajiboye, 2016).

The transition to online learning may thus have impacted students differently, potentially allowing some to prosper while leaving others adversely affected. More information about translation students’ self-perceptions and coping strategies in the wake of the COVID-19 pandemic and the resulting hastened transition to online learning is therefore needed to better inform educators about the best teaching practices and learning environments. A potential factor influencing how students react to this transition to online learning could be self-efficacy.

### The Translation Self-Efficacy Framework

In psychological terms, self-efficacy is defined as, “the belief in one’s capability to execute required actions and produce outcomes for a defined task” (Wood et al., 2000). The social-psychological framework has been applied to translation studies by examining the processes and features of translators as “bilingual agents” and social individuals in translation marketplaces within socio-cultural contexts (Risku et al., 2017; Zhu, 2020). Although the concept of self-efficacy has been the subject of much psychological research dating back to the 1970’s, it has only recently been used in the domain of translation studies. Bolaños-Medina (2014) and Mashhady et al. (2015) were among the first to incorporate it into such research.

According to Haro-Soler (2019), there are two components of self-efficacy. First, the individual demonstrates self-efficacy as the ability to carry out a particular task. Second, self-efficacy beliefs are perceptions of one’s ability to perform a specific task (Bandura, 1986; Haro-Soler, 2019). Within the translation self-efficacy literature, self-efficacy beliefs are distinguished from self-efficacy to reflect a translator’s confidence in one’s ability to translate a particular text in specific situational circumstances and conditions. For example, these conditions may function differently depending on the type of assignment within a translation course (Haro-Soler, 2019). As such, researchers conclude that self-efficacy is important for tolerating ambiguity, achieving success in process-oriented studies, and comprehending advanced source language reading comprehension (Bolaños-Medina, 2014; Mashhady et al., 2015; Bolaños-Medina and Núñez, 2018).

Linnenbrink and Pintrich (2003) posit that self-efficacy plays a large role within the domains of commitment, effort, and behavior, wherein students lacking sufficient self-confidence are less likely to put in effort and more likely to abandon a task without finishing it. Additionally, those with low self-confidence in such areas may also internalize a belief that they do not have the necessary skills and characteristics to achieve mastery through their own actions, known as “learned helplessness” (Linnenbrink and Pintrich, 2003; Mashhady et al., 2015). Researchers have studied the marker of general self-efficacy, as it may be used as a universal construct (Schwarzer and Jerusalem, 1995; Scholz et al., 2002). Many studies have shown its usefulness empirically as a universal construct that can be practically measured within a one-dimensional model (Schwarzer and Jerusalem, 1995; Scholz et al., 2002; Scherbaum et al., 2006). The General Self-Efficacy Scale (GSE) was pioneered by Schwarzer and Jerusalem (1995), wherein higher scores have been successful in predicting work-satisfaction, optimism, and positive emotional affect. On the other hand, lower GSE scores predict burnout, stress, depression, physiological health issues, and anxiety (Schwarzer and Jerusalem, 1995). As such, the role of self-efficacy has also been examined in translation studies to elucidate self-beliefs of students and predict future behaviors.

Formal programs seeking to develop translation skills, often decentralized in nature, have broad systems of instruction and training programs (Kelly, 2008; Haro-Soler, 2019; Haro-Soler and Kiraly, 2019). Haro-Soler (2017) argued that having greater self-efficacy beliefs is important for translators in developing their skills and achieving educational goals. Empirical studies have attempted to identify ways to enhance the development of self-efficacy and self-perception beliefs among translation students (Yang et al., 2016, 2021; Haro-Soler, 2017, 2019; Haro-Soler and Kiraly, 2019). Haro-Soler (2019) posits that translation teachers and students should leverage the “social construction of knowledge” and focus less on teacher-centered education. Skills related to one’s confidence to work independently may lead translation students to obtain the necessary research skills through increased self-esteem and self-efficacy (Haro-Soler, 2019).

Researchers have used the learner-centered, social-constructivist approach to examine the construction of translation knowledge both among translation teachers and students. With the greater integration of technological innovations in education however, more focus has been paid to topics such as machine translation, which are now ubiquitous among translators. As such, Haro-Soler (2019) argues that translators may be able to complete these processes with computerized tools and machine translation only in the future. From the social constructivist viewpoint however, it is assumed that the development of practical knowledge and theories involving translation and research are still essential within any related training program.

According to Ferreira and Schwieter (2017), there is an association between general self-efficacy and the ability to complete complex tasks specific to translation. They state that perceptions of difficulty in understanding source texts, editing, and the ability to handle high cognitive loads can benefit translation. Bolaños-Medina (2014) examined self-efficacy in its association with translatology, sociology, and cognition, finding that self-efficacy is valuable within translation processes for the purpose of managing ambiguity, documentation activities, translation process studies, and comprehending source languages. Mashhady et al. (2015) found self-efficacy was important for predicting note-taking inclinations among undergraduate translation students, an activity they regarded as a personal strategy to cope with challenges of interpretation.

The theoretical perspective of self-efficacy can be broken down into four parts. These areas include *enactive mastery experience, vicarious experience, verbal persuasion, and physiological and emotional state* (Bandura, 1977).

*Enactive mastery experiences* refer to the leveraging of past successes in one’s ability when executing a task to increase positive self-perceptions (Zhang and Ardasheva, 2019; Yang et al., 2021). As such, translation students base their self-perceived abilities to achieve a goal on their past experiences. A high degree of self-efficacy may be obtained by analyzing past experiences in which one has succeeded at a translation task and overcome obstacles concerning their work. When students feel they have mastered a task, it subsequently creates a powerful sense of trust in their own abilities (Bandura, 1977; Yang et al., 2021).

*Vicarious experiences* describe an individual’s observation concerning their own or others’ past successes and failures (Schunk and Hanson, 1985). Observing other translators or translation students may thus give students more confidence in their ability to pursue their own goals with a greater degree of self-efficacy.

*Verbal persuasion* refers to a reflection consisting of verbal feedback regarding performance abilities. As such, verbal persuasion is used to examine affective states, effort, and task choice in relation to self-efficacy (Bandura, 1977). Statements about a student’s skills rather than underperformance on translation tasks may raise self-efficacy.

Finally, *physiological and emotional states* describe translation students’ reactions in relation to managing emotional and physical stressors arising when performing translation tasks. Physiological and emotional factors influencing self-efficacy include engagement with a highly complex task, as well as being in a depressed, anxious, or stressed state of mind (Bandura, 1977).

Bandura (1977) further posits that there are three dimensions of self-efficacy belief. These three dimensions include *magnitude, strength, and generality*.

*Magnitude* describes the level of difficulty translation students perceive regarding a particular task (Bandura, 1977). For example, individuals may have low self-efficacy beliefs when the task is thought to be more difficult, a high magnitude task. On the other hand, students may experience high self-efficacy beliefs when completing a low-magnitude task (Bandura, 1977).

*Strength* refers to a student’s judgment about their abilities to complete a particular translation task (Bandura, 1977). A student with a high belief in their own capacity to complete a particular task may thus behave in ways that increase their persistence and diligence, while also decreasing the frustrations associated with obstacles inherent to the task (Bandura, 1977).

Finally, *generality* describes an individual’s confidence their abilities may be generalized more broadly from one task to another. When an individual has high self-efficacy generality, they believe if they do well in one task, they can generalize such abilities to perform well in a variety of other tasks (Bandura, 1977).

### Self-Efficacy and Achievement in Translation Studies

Translation students reported that the most important factor influencing personal trust in their own translation abilities was the belief that they are able to produce adequate translations (Haro-Soler, 2017). Self-efficacy, which is associated with the belief that they will be able to meet a self-directed goal associated with a particular task, allows translators to justify their own decisions. The literature on self-efficacy and material achievement in translation studies is however very brief. Mashhady et al. (2015) examined self-efficacy in terms of predicting note-taking inclinations among undergraduate translation students, including at both the junior and senior levels, in the University of Zabol, Iran. The author found a positive correlation between GSE scores and note-taking tendencies. Specifically, the tasks included noting “ideas links, negation, emphasis, verticality, and shift” (Mashhady et al., 2015, p. 2366). The results also showed similar scores for both female and male students in self-efficacy, although the self-efficacy of male students (27.27) was higher than that of females (25.84). There were no significant differences however between females and males in note-taking inclinations. These findings support those of Angelelli and Jacobson (2009) and Monacelli (2009) showing no significant differences between females and males in interpreting sub-skills.

### Translation, Cognition, and Decision-Making

Achievement in translation studies not only depends on the social-psychological framework, but also the cognitive framework. Shih (2015) was the first to examine decision making and problem-solving strategies as fundamental processes in translation research. The author states that the six stages of decision making involve: (1) problem identification; (2) problem clarification (description); (3) research on, and collection of, background information; (4) deliberation on how to proceed (pre-choice behavior); (5) the moment of choice; and (6) post-choice behavior (the evaluation of translation results) (p. 2). More recently, researchers have examined translation studies beyond broad linguistic activity. They have come to recognize that translation is also a highly complex cognitive task. As such, cognitive factors have helped us understand the study of translation in the form of problem solving and puzzle solving activities, as well as fine sensory-motor abilities (Rojo et al., 2014; Rojo, 2015). The various cognitive elements involved therefore make these processes exceptionally difficult to master and thus remain mostly unexplained.

Obdrzalkova (2016) posits that translation is a decision-making process wherein the translator processes a series of decisions by choosing one of many alternative options. When selecting the most optimal choice, they must narrow down the list of various alternatives which have different semantic, stylistic, and rhythmical features. In the area of technological innovation, their work is the subject of scholarly debate, as there is no single computer program that can create a highly accurate translation. Although such technology has been highly useful in areas such as STEM sciences, no technological innovation thus far has managed to create a faultless translation output, making this line of research even more challenging (Rojo, 2015).

Translation requires a high degree of mental concentration, allowing us to view the activity as one of mental stimulation. In practical teaching settings, Gorozhanov et al. (2018) explain how the trust between an online translation teacher and the student is dependent upon an understanding of the teacher as an expert providing correct suggestions about one’s cognitive processes and work. Specifically, the teacher must command a strong vocabulary in the source and the target language. The aspiring translator must become cognizant of stylistic variations surrounding grammatical forms, synonyms, and syntactic structures. The teacher must have contextual knowledge regarding the social and cultural information that is encoded and transmitted, recognizing both implicit as well as explicit meanings (Gorozhanov et al., 2018).

Since it is unlikely that a fully automated translation program will be able to accurately complete such tasks in the near future, more research is needed on the nature of constructing meaning within the cognitive framework itself (Rojo, 2015; Obdrzalkova, 2016). In understanding the psychological and cognitive processes of translations, researchers must view the construction of meaning as fundamentally embodied, situated, and dynamic phenomena. Rojo (2015, p. 722) claims that “*meaning is embodied* means that it is grounded in the way human beings use their bodies to interact with the world.” As such, people construct meaning in a grounded capacity, similar to the way in which humans interact physically with their environment and social contexts.

Within the so-called Embodied Simulation Hypothesis for example, translation includes mentally stimulating actions and perceptions that are tied to the external domain (Rojo, 2015). This suggests language construction involves the activation of social, motor, perceptual, and knowledge-based processes, greatly impacting those involved when translating. Within the Embodied Simulation Hypothesis, translators are actors in charge of constructing and reconstructing meaning between the original content, communicating this to a particular audience with the appropriate linguistic labels (Rojo, 2015; Obdrzalkova, 2016). Translators may also be highly constrained by environmental factors, such as “restrictions of the textual content, those of the working environment, or the prevailing norms from the cultural and historical context” (Rojo, 2015, p. 723). These factors inform the translator’s mental experiences *via* their base knowledge, ideological preferences, individual personalities, and individual behaviors. Núñez and Bolaños-Medina (2018) state that those involved in translation interact with social and technical environments, along with their own personal history and other influences.

The technical environment and resulting influence are therefore enmeshed within causal, process, and comparative models. Núñez and Bolaños-Medina (2018) for example used an empirical approach to examine translators’ self-perceived problem-solving self-efficacy. They found that competence, intrinsic motivation toward accomplishment, and the self-perceived problem-solving efficacy of translation students were interrelated. Specifically, their research demonstrated a linear relationship between the aforementioned variables and that intrinsic motivation toward accomplishment and competence are significant predictors of self-perceived problem-solving efficacy, explaining 39% of the variance (Núñez and Bolaños-Medina, 2018). As such, Rojo (2015) asserts that clarifying the relationship between cognitive linguistic processes and psychology may give way to new experiments that could further close the knowledge gap concerning language processing, communication, and translation (Rojo, 2015).

### Computer Self-Efficacy

Bandura (1977) pioneered research in computer self-efficiency, an area defined as a self-perception of one’s abilities to use a computer (Compeau and Higgins, 1995). According to Kinzie et al. (1994), those who have high self-efficacy with computers are more likely and willing to use a computer to learn. Computer self-efficacy has been studied in education among both students (Aşkar and Umay, 2001; Akkoyunlu and Kurbanoğlu, 2003 and teachers (Özçelik and Kurt, 2007). Additionally, those with positive attitudes about computers are more likely to have higher general self-efficacy (Kinzie et al., 1994). Computer self-efficacy is also associated with the decision to increase personal computer usage in several studies (Hakverdi et al., 2007). To better understand levels of computer self-efficacy, Aşkar and Umay (2001) created the Computer Self-Efficacy Scale (CSE), which is frequently used in education research. When studying a sample of teaching students in Turkey, Akkoyunlu and Kurbanoğlu (2003) found that pre-service teachers in science, mathematics, and biology showed a low level of familiarity with computers after being asked to report their perceptions of computer self-efficacy through the CSE (Aşkar and Umay, 2001; Akkoyunlu and Kurbanoğlu, 2003; Yilmaz et al., 2006). Using the same scale (Aşkar and Umay, 2001), Topkaya (2010) found that general self-efficacy predicted computer self-efficacy among teachers, although there was a moderate and positive correlation between the two factors. As such, it may be valuable to understand how well this scale is able to predict outcomes among translation students.

### Computer-Based Translation Tools

Technological developments in translation have given way to the increasingly widespread use of Computer-Assisted Translation (CAT) tools (Odacioglu and Kokturk, 2015). Pietrzak and Kornacki (2021) for instance have found that 63.5% (90 participants) of professional translators use CAT tools in a sample of 141 respondents. Two-thirds of them were below the age of 50, while over two-thirds had at least 10 years of translation experience. Participants aged 60 and over were the least likely to use CAT. As such, younger translators would appear to be more comfortable using CAT tools (Pietrzak and Kornacki, 2021). Additionally, 44 of respondents (49.8%) claimed they use more than one CAT tool at work; 26 of CAT users (28.9%) use two tools; 10 of CAT users (11.1%) use three tools; while 3 CAT users (3.33%) reportedly use at least five tools. The most common tool was SDL Trados Studio. Other tools used included Across, Déjà vu, Omega T., Open Language Tools, MateCat, Star Transit, Wordbee, Memsource, and SmartCat. Although widely used, only 40% of respondents felt such resources were of good quality and 90% encountered technical issues with CAT computer compatibility (Pietrzak and Kornacki, 2021).

The necessary computer skills in this area have changed drastically as new technologies become increasingly integrated into academic translation teaching and learning. Among translation students, the incorporation of CAT tools has necessitated novel skills, such as localization engineering which involves translation for websites, e-learning modules, and software applications (Odacioglu and Kokturk, 2015). The use of applications such as Nubuto for example, an online resource, appears to benefit students even before fully realizing the translation process. There is a need however for translation tools to become more “student oriented” in combining theory and practice (Odacioglu and Kokturk, 2015). There may nevertheless be inherent discomforts surrounding technology and anxiety due to cognitive friction, the frustration experienced following unexpected outcomes (Pietrzak, 2020). Cognitive friction in this context occurs when “unhelpful or distracting CAT tool features” result in an extraneous cognitive load (O’Brien et al., 2017). Throughout the COVID-19 pandemic, a greater reliance has been placed upon technology, significantly affecting translation activities. As such, it has become increasingly difficult for students to deal with such challenges due to gaps in existing technological skills (Pietrzak, 2020).

### Gaps in the Literature

In summary, existing research appears to suggest the widespread transition to online learning following the COVID-19 pandemic may have negatively impacted the learning experience of students. This appears to be particularly true for translation students, a field requiring a greater degree of face to face interaction in order to convey the necessary implicit knowledge regarding specific language subtleties and context. It also seems some students may have experienced more negative affects as a result of this transition than others.

This inevitably raises the question then, what could be the underlying cause behind such a differential experience among translation students? Existing research would suggest that those with high levels of self-efficacy are more likely to possess the underlying traits allowing them to thrive in an independent learning environment, including setting personal goals and not giving up in the face of adversity. No study to date however has examined the direct effects of self-efficacy in determining student achievement in translation or other outcomes such as computer self-efficacy, a void this research seeks to fill.

## Aims of the Study

The purpose of this study is to examine the effects of self-efficacy and computer self-efficacy on academic success among undergraduate translation students. The study also aims to investigate the role of self-efficacy and computer self-efficacy in typing frequency among the target group before and after the commencement of the COVID-19 pandemic.

As such, three hypotheses are advanced here. Hypothesis 1 states that general self-efficacy will predict computer self-efficacy. Hypothesis 2 states that (a) general self-efficacy will predict GPA among undergraduate students, and (b) computer self-efficacy will predict GPA over and above general self-efficacy. Hypothesis 3 states that (a) general self-efficacy will predict reported changes in computer typing frequencies (before and after the start of the COVID-19 pandemic), and (b) computer self-efficacy will predict changes in typing frequencies over and above general self-efficacy.

By testing these hypotheses, we may gain a better understanding of the factors contributing to improved academic performance among translation students in remote, online learning settings.

## Methodology

### Instruments

To examine the effects of general and computer self-efficacy on translation learning, senior Bachelor of Arts students from the English Language Department of Umm Al-Qura University, were sent a URL for a survey that included the GSE questionnaire (Schwarzer and Jerusalem, 1995), the CSE questionnaire (Aşkar and Umay, 2001), and a questionnaire regarding demographic factors and GPA. Eighty-three (10%) undergraduate translation students (31 female, 52 male) participated in the study.

The GSE measures self-perceived self-efficacy. It consists of 10 items on a four-point Likert scale, with the total score varying from 10 to 40 points. Schwarzer and Jerusalem (1995) reported internal reliability analysis revealed a Cronbach’s Alpha Coefficient of 0.88, demonstrating a high internal consistency. The answers correspond to a four-point Likert-like scale, including: (1) Not at all true; (2) Hardly true; (3) Moderately true; (4) Exactly true.

The CSE consists of 18 items measuring computer affect, including values, self-judgments, and attitudes. This study included 13 items relevant to translation students. The reasoning behind this was that Schwarzer and Jerusalem (1995) used only positive valence items, while Aşkar and Umay (2001) used a mixture of positive and negative valence items. To maintain compatibility between the two scales, only positive valence items in the CSE (Aşkar and Umay, 2001) are used here. Aşkar and Umay (2001) reported a Cronbach’s Alpha Coefficient of 0.71, demonstrating high internal consistency. The answers for the CSE are similar to the GSE which uses a five-point scale. For the purposes of this study however, the CSE was used with a four-point scale to align with the GSE, including the anchors: (1) Not at all true; (2) Hardly true; (3) Moderately true; (4) Exactly true.

Students also reported their overall GPAs on the university scale of 1–6, with 1 being the lowest, and 6 being the highest possible. Participants were also asked to specify their gender with the options Male or Female. To examine typing frequency before and after the pandemic, they answered two items. The first was (1) “How would you rate your typing skills on a scale of 1 to 10 before the pandemic started?” The second was (2) “How would you rate your typing skills on a scale of 1 to 10 after the pandemic started?” The differences between the second and first scores were calculated to measure the possible change of typing skills, implying changes in typing frequencies following the beginning of the pandemic. This difference was then computed as the dependent variable, “change in typing frequencies.”

### Analysis of the Data

The data was obtained using the SurveyMonkey software tool and analyzed with the use of the SPSS 28 analytics platform. Descriptive analyses, correlation analyses, one-way ANOVAs, and hierarchical linear regression analyses were conducted to further examine the research hypotheses. The choice of statistical tests was based on the research hypotheses.

## Results

Among the undergraduate translation students, the mean of the GSE, measuring general self-efficacy, stood at 2.88 (SD = 0.62) out of a maximum of 4. As seen in Table 1, comparable to the findings of Schwarzer and Jerusalem (1995) with an alpha of 0.88, the GSE had a high level of internal consistency with an alpha of 0.893.

**TABLE 1:** Distribution of answers of the general self-efficacy scale (*N* = 83).

No.	Item	M	SD
1	I can always manage to solve difficult problems if I try hard enough.	3.32	0.80
2	If someone opposes me, I can find the means and ways to get what I want.	2.92	0.86
3	It is easy for me to stick to my goals.	2.68	0.99
4	I am confident that I could deal efficiently with unexpected events.	2.62	0.88
5	Thanks to my resourcefulness, I know how to handle unforeseen situations.	2.48	0.93
6	I can solve most problems if I invest the necessary effort.	3.35	0.74
7	I can remain calm when facing difficulties because I can rely on my coping abilities.	2.58	0.96
8	When I am confronted with a problem, I can usually find several solutions.	2.91	0.83
9	If I am in trouble, I can usually think of a solution.	3.14	0.86
10	I can usually handle whatever comes my way.	2.72	0.88
	General self-efficacy	2.88	0.62

The second scale measured computer self-efficacy (CSE). Translation students demonstrated a mean score of 2.83 (SD = 0.64) out of a maximum of 4. The CSE also maintained a high level of reliability with an alpha coefficient of 0.897, as seen in Table 2. The alpha level (0.897) of the adapted CSE reported in the current study is higher than that reported by Aşkar and Umay (2001) at 0.71. This shows how the adapted version of the CSE, not including negative valence items, has a much higher degree of internal reliability than the original measure.

**TABLE 2:** Distribution of answers of the computer self-efficacy scale (*N* = 83).

No.	Item	M	SD
1	If I try hard, I can solve the problems related to computers.	3.02	0.94
2	I think I can use the computer efficiently.	3.20	0.79
3	I surf in the computer and make new discoveries	3.01	1.04
4	It is easy for me to write all kinds of things on the computer.	3.12	0.94
5	I am talented about computers.	2.83	0.97
6	I feel competent when computers are concerned.	2.98	0.84
7	At-the-moment solutions while working with computers are enough for me.	3.00	0.82
8	I know what to do when I meet a new thing while working with computers.	3.04	0.85
9	I believe that I master computer terminology and concepts.	2.60	1.05
10	I think of computers almost as a part of me.	2.67	1.08
11	I believe I have a special talent toward using computers	2.44	1.07
12	I have always believed that it is impossible for me to master computers totally	2.50	1.10
13	I use computers while planning my day.	2.39	1.14
	Computer self-efficacy	2.83	0.64

### Regression Analyses

A regression analysis showed that computer self-efficacy was a significant predictor of general self-efficacy, Δ*R*^2^ = *0.33*,Δ*F*(1,80) = 40.91, *p* < 0.001. As general self-efficacy increased, computer self-efficacy increased significantly, β = *0.57*, *p* < 0.001, 95% CI (0.39, 0.75). Additionally, the results from an analysis of variance showed that gender was a significant predictor of GPA, *F*(1,82) = 18.37, as female students demonstrated higher averages (*M* = 4.70, SD = 0.32) than male students (*M* = 3.48, SD = 1.22) when it came to GPA (1–6 scale). The results of the CSE analysis (*M* = 2.5) indicate students were comfortable using computers. There were however no significant differences between males and females on computer self-efficacy. Similarly, the mean values for items 1 and 2, *M* = 3.02 (SD = 0.94) and 3.20 (SD = 0.79), respectively, support the idea that students possess moderate self-efficacy perceptions regarding computers. Given that none of the items’ mean values lie closer to 4, it can be inferred that the sample population is not highly confident in terms of computer usage. Likewise, the mean value of item 13, “I use computers while planning my day” (*M* = 2.39, SD = 1.14) supports the idea that computers are not an integral part of translation students’ planning activities (Table 3).

**TABLE 3:** Means, standard deviations, range, bivariate correlations, and Cronbach’s alphas of predictor and dependent variables.

Variable	*N*	*M* (SD)	Range	1	2	3	4	α
1. Gender (Male)	83		1–2					
2. GSE	83	2.88 (0.62)	1–4	–0.09				0.893
3. Computer SE	83	2.83 (0.63)	1–4	–0.01	0.58**			0.897
4. GPA	83	3.93 (1.39)	1–6	–0.43**	0.40**.	0.18		
5. Change Type	83	3.93 (1.39)	1–10	0.07	0.21*	0.06	0.17	

*p-value is less than 0.05 and **correlation is significant at the 0.01 level (two-tailed).

**FIGURE 1:**
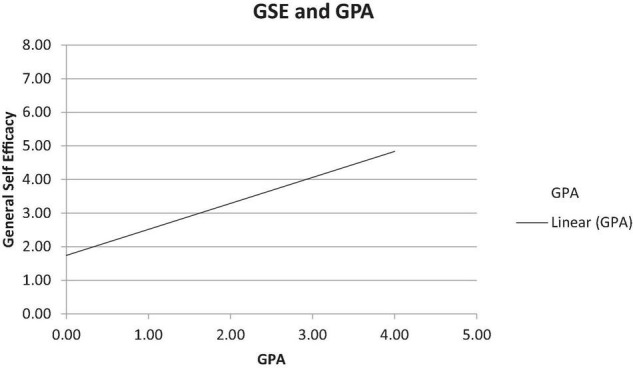
Linear relationship between GPA and general self-efficacy.

A hierarchical regression model with bootstrapping was used to determine the predictive value of the independent variables (Gender, GSE, Computer SE) and GPA. Gender was added in Step 1, GSE was added in Step 2, and Computer SE was added in Step 3, as seen in Table 4. Bootstrapping was performed using 2,000 samples with 95% bias-corrected and accelerated (BCa) confidence intervals. The regression parameters reflected 95% confidence interval estimates. All the Tolerance values were greater than 0.1 and all the Variance Inflation Factor (VIF) values were below 10. In Step 1, the results demonstrated that gender predicts GPA, Δ*R*^2^ = *0.*18, Δ*F*(1,80) = 17.76, *p* < 0.001, as female students possessed higher GPAs than male students, β = *–0.43*, *p* = 0.001, BCa CI (0.61, 1.80). Interestingly, the hierarchical linear regressions indicated that general self-efficacy predicts GPA over and above gender, as seen in Figure 1.

**TABLE 4:** Summary of hierarchical regression for the prediction of GPA.

							95% BCa CI					
							
Step	Predictor	*B*	SE	β	*t*	*p*	*Lower*	*Upper*	*R* ^2^	Δ*R*^2^	df	Δ*F*
1.	Gender	–1.21	0.29	–0.43	–4.2	0.001	0.61	1.80	0.18	0.18	1,80	17.76
2.	GSE	–0.81	0.21	–0.36	–3.8	0.001	0.43	1.15	0.31	0.13	1,79	14.66
3.	Computer SE	0.11	0.25	05	0.44	0.66	–0.62	0.40	0.31	0.01	1,78	0.91

*N = 83. CI, Confidence Intervals.*

*p < 0.05.

The results demonstrate that gender and general self-efficacy predict GPA among undergraduate translation students. The results of Step 2 were significant, Δ*R*^2^ = *0.13*,Δ*F*(1,79) = 14.66, *p* < 0.001, as general self-efficacy predicted GPA over and above gender, β = *–0.36*, *p* < 0.001, 95% BCa CI (0.43, 1.15). It was anticipated that computer self-efficacy would predict GPA over and above gender and general self-efficacy. The results of Step 3 were not significant, Δ*R*^2^ = *0.01*,Δ*F*(1,78) = 0.19, *p* = 0.66, as computer self-efficacy did not predict GPA over and above gender and general self-efficacy, β = *0.05*, *p* = 0.66, BCa CI (–0.62, 0.40).

**FIGURE 2:**
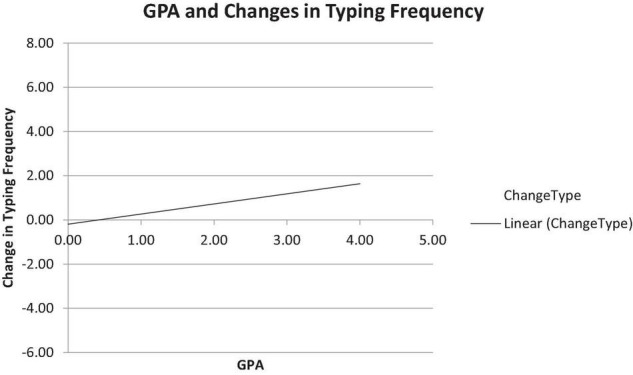
A linear relationship between GPA and typing frequency change following commencement of the COVID-19 pandemic.

General self-efficacy and computer self-efficacy were anticipated to predict computer usage after the COVID-19 quarantine had commenced. A regression analysis revealed that general self-efficacy predicted an increase in reported typing frequency after compared to before the COVID-19 pandemic began, Δ*R*^2^ = *0*.045, Δ*F*(1,79) = 3.80, *p* < 0.05, as general self-efficacy predicted an increase in overall computer typing frequencies, β = 0*.67*, *p* = 0.001, 95% CI (–2.80, 1.20). As mentioned above, a hierarchical regression model was run to determine the predictive value of the independent variables and the dependent variable as part of three steps. Interestingly, the results of Step 2 show a significant effect for self-efficacy and typing frequency, Δ*R*^2^ = *0*.05, Δ*F*(1,79) = 4.0, *p* = 0.05, as general self-efficacy predicted a change in typing frequency after the beginning of the COVID-19 quarantine, β = –0.22, *p* = 0.05, 95% CI (–0.01, 1.55), as seen in Figure 2.

However, typing frequency was not significantly predicted by gender or computer self-efficacy, as seen in Table 5.

**TABLE 5:** Summary of hierarchical regression for increased computer typing following commencement of the COVID-19 pandemic.

							95% CI					
							
Step	Predictor	*B*	*SE*	β	*t*	*p*	*Lower*	*Upper*	*R* ^2^	Δ*R*^2^	df	Δ*F*
1.	Gender	0.26	0.44	0.07	0.59	0.59	–1.11	0.65	0.01	0.01	1,80	0.35
2.	GSE	0.68	0.34	–0.22	2.0	0.05	–0.01	1.55	0.05	0.05	1,79	4.0
3.	Computer SE	–0.30	0.41	0.10	–0.73	0.47	–1.07	0.53	0.06	0.01	1,78	0.53

*N = 83. CI, Confidence Intervals.*

*p < 0.05.

## Discussion and Conclusion

After the commencement of the COVID-19 pandemic, educational institutions all over the world witnessed the widespread cessation of face to face instruction. For translation studies, direct instruction between lecturers and students is especially important in providing the practical skills needed to complete translation tasks. Technological advancements in online learning have become ubiquitous in recent years. Specifically, student-centered instruction techniques are offered online *via* technological tools for sharing, transferring, and extending knowledge, while access to academic services and resources is also provided as well (Almahasees and Qassem, 2021). Researchers however have noted that many students in Saudi Arabia do not view online learning as a replacement for face to face instruction (Al-Jarf, 2020). Although many fields, such as STEM disciplines, may rely greatly on computer programs to foster various academic outcomes, this is arguably not feasible for translation studies, as no technological innovation thus far has provided consistently accurate translation outputs. Understanding translation students’ self-perceptions, including self-efficacy, may provide a better understanding of how to best harness the social-psychological features that may improve performance and resourcefulness in an online learning environment.

This study has investigated the roles of self-efficacy and computer self-efficacy in predicting GPA and translation students’ grades. Bandura (1977) first advanced the idea of self-efficacy as an individual’s belief in their own ability to successfully produce optimal outcomes for a designated task. Although the literature on self-efficacy and translation studies is relatively young, research thus far suggests higher levels of self-efficacy is an important trait among high achieving translation students. Supporting these findings, the results reported here reveal an important role for self-efficacy and computer self-efficacy in predicting GPA and translation course grades.

Two scales were used in this study. The first included the GSE scale measuring general self-efficacy, the results of which demonstrated a high level of internal consistency with an alpha of 0.893. The second included the CSE scale measuring computer self-efficacy, also showing a high level of internal consistency with an alpha of 0.897. The findings indicated that general self-efficacy, after controlling for gender, significantly predicts GPA among undergraduate translation students. General self-efficacy also predicted increased typing frequencies after the commencement of the COVID-19 quarantine. It is possible therefore that students with higher self-efficacy fare better in remote learning environments, which may be reflected in increased computer usage.

The alternative proposition for Hypothesis 1 was confirmed, as General self-efficacy was a significant predictor of Computer self-efficacy. Hypothesis 2 was partially supported. While (a) General self-efficacy significantly predicted GPA, (b) Computer self-efficacy did not predict GPA over and above Gender and General self-efficacy. Also, the results showed significantly higher GPAs among female compared to male undergraduate translation students. It appears that the variance in General self-efficacy and Computer self-efficacy on GPA, after controlling for gender, is somewhat shared. These results support those of several studies investigating the role of self-efficacy in determining translation studies achievement (Mashhady et al., 2015; Haro-Soler, 2017, 2019; Núñez and Bolaños-Medina, 2018; Yang et al., 2021). As such, future researchers should further examine the ways in which Computer self-efficacy can be seen as distinct from General self-efficacy.

Hypothesis 3 was also partially supported. Specifically, (a) General self-efficacy significantly predicted changes in typing frequency before and after the commencement of the COVID-19 pandemic. Interventions designed to enhance general self-efficacy among translation students may be an important tool to help increase computer usage during the pandemic, which in turn may enhance students’ abilities to gain a better footing when learning translation online. This study’s findings support the need for such interventions, as increased typing frequency following the beginning of the pandemic was positively associated with general self-efficacy (*r* = 0.21, *p* = 0.05). As general self-efficacy increased, computer self-efficacy increased significantly, β = *0.57*, *p* < 0.001, 95% CI (0.39, 0.75). The results also showed that (b) computer self-efficacy did not predict changes in typing frequencies over and above general self-efficacy. As such, similarly for Hypothesis 2, there appears to be shared variance between the two factors. Based on this study’s findings, it could be concluded that interpreting learners’ self-efficacy can help them to overcome their doubts when choosing appropriate learning strategies in a digital environment. Enhancing students’ self-efficacy may therefore improve their ability to digest learning material and solidify appropriate online study habits.

This research supports findings from Almahasees and Qassem (2021) who concluded online learning requires independent critical thinking and self-direction. These attributes are thought to be one of the most prominent features setting student-centered and teacher-centered learning apart. Since general self-efficacy predicted increases in typing frequency following the commencement of the pandemic, it appears beneficial online learning strategies include higher levels of self-perception about one’s own behavioral learning actions, processes, and self-knowledge. The self-perceptions associated with self-efficacy particularly appear to improve learning indicators, including typing frequency, in online learning environments for translation students.

Since there were no significant results associated with computer self-efficacy above and beyond gender and general self-efficacy, general self-efficacy appears to be the most important marker for GPA among translation students. Additionally, general self-efficacy had the effect of increasing typing frequencies and thus computer usage for translation studies. As such, these data suggest students with greater general self-efficacy are more likely to use technology efficiently during times in which in-person translation lessons are unavailable. Future research could therefore focus on the differences in self-efficacy among students who believe online learning is helpful versus those who believe it is unhelpful and significantly challenging.

The study’s limitations include the use of the CSE, as it appeared to share variance to a significant extent in predicting GPA and changes in typing frequency before and after the start of the COVID-19 pandemic. Since general self-efficacy greatly predicted computer self-efficacy however, it is possible that these factors are simply robust, necessary features explaining academic performance among undergraduate translation students in an online learning environment. A possible reason for this finding may be associated with the Computer Self-Efficacy Scale itself, as it was published in 2001 (Aşkar and Umay, 2001). Future research should thus examine updated methods for measuring computer self-efficacy in a way that aligns with more recent uses of technology in translation studies. Future research should also investigate whether general self-efficacy and computer self-efficacy predicts the belief online learning is helpful or unhelpful, as this could provide greater clarity concerning the utility of facilitating adaptive resources to increase computer self-efficacy among translation students.

## Data Availability Statement

The original contributions presented in the study are included in the article/supplementary material, further inquiries can be directed to the corresponding author.

## Ethics Statement

Ethical review and approval was not required for the study on human participants in accordance with the local legislation and institutional requirements. The patients/participants provided their written informed consent to participate in this study.

## Author Contributions

The author confirms being the sole contributor of this work and has approved it for publication.

## Conflict of Interest

The author declares that the research was conducted in the absence of any commercial or financial relationships that could be construed as a potential conflict of interest.

## Publisher’s Note

All claims expressed in this article are solely those of the authors and do not necessarily represent those of their affiliated organizations, or those of the publisher, the editors and the reviewers. Any product that may be evaluated in this article, or claim that may be made by its manufacturer, is not guaranteed or endorsed by the publisher.

## References

[B1] Akkoyunlu B. Kurbanoğlu S. (2003). A study on teacher candidates’ perceived information literacy self-efficacy and computer self-efficacy. *Hacettepe Univ. J. Educ.* 24 1–10.

[B2] Albion P. R. (1999). “Self-efficacy beliefs as an indicator of teachers’ preparedness for teaching with technology,” in *Proceedings of SITE 1999–Society for Information Technology & Teacher Education International Conference*, eds Price J. Willis J. Willis D. Jost M. Boger S. (Waynesville, NC: Association for the Advancement of Computing in Education (AACE)), 1602–1608.

[B3] Albion P. R. (2001). Some factors in the development of self-efficacy beliefs for computer use among teacher education students. *J. Technol. Teach. Educ.* 9 321–347.

[B4] Al-Jarf R. (2020). Distance learning and undergraduate Saudi students’ agency during the Covid-19 pandemic. *Bull. Trans. Univ. Brasov, Ser. IV Philol. Cult. Stud.* 13 37–54. 10.31926/but.pcs.2020.62.13.2.4

[B5] Almahasees Z. Qassem M. (2021). Faculty perception of teaching translation courses online during Covid-19. *PSU Res.Rev.* 5 1–15. 10.1108/PRR-12-2020-0044

[B6] Alves F. Pagano A. da Silva I. A. (2010). “A new window on translators’ cognitive activity: methodological issues in the combined use of eye tracking, key logging and retrospective protocols”. in *Methodology, Technology and Innovation in Translation Process Research: a Tribute to Arnt Lykke Jakobsen*, eds Mees I. M. Alves F. Göpferich S. (Frederiksberg: Samfundslitteratur), 267–291.

[B7] Alwazna R. Y. (2021). Teaching translation during COVID-19 outbreak: challenges and discoveries. *Arab World Engl. J. (AWEJ)* 12 86–102. 10.24093/awej/vol12no4.6

[B8] Angelelli C. V. Jacobson H. E. (2009). “Testing and assessment in translation and interpreting studies: A call for dialogue between research and practice”, in *Testing and Assessment in Translation and Interpreting Studies*, eds Angelelli C. V. Jacobson H. E. (Amsterdam: John Benjamins Publishing Company), 1–10.

[B9] Aşkar P. Umay A. (2001). Perceived computer self-efficacy of the students in the elementary mathematics teaching programme. *Hacettepe Univ. J. Educ.* 21 1–8.

[B10] Bandura A. (1977). Self-efficacy: towards a unifying theory of behavioural change. *Psychol. Rev.* 84 191–215. 10.1037/0033-295X.84.2.191 847061

[B11] Bandura A. (1986). *Social Foundations of Thought and Action: A Social Cognitive Theory.* Hoboken, NJ: Prentice-Hall.

[B12] Bolaños-Medina A. (2014). Self-efficacy in translation. *Transl. Interpret. Stud.* 9 197–218. 10.1075/tis.9.2.03bol 33486653

[B13] Bolaños-Medina A. Núñez J. L. (2018). A preliminary scale for assessing translators’ self-efficacy. *Across Lang. Cult.* 19 53–78. 10.1556/084.2018.19.1.3

[B14] Chiluwa I. Ajiboye E. (2016). “Language use in crisis situations: a discourse analysis of online reactions to digital news reports of the Washington navy yard shooting and the nairobi westgate attack.” in *The Discourse of Digital Civic Engagement: Perspectives from the Developing World*, eds Taiwo R. Opeibi T. (New York, NY: Nova Science Publishers), 35–55

[B15] Compeau D. R. Higgins C. A. (1995). Computer self-efficacy: development of a measure and initial test. *MIS Q.* 19 189–211. 10.2307/249688

[B16] Eggen P. D. Kauchak D. P. (2007). *Educational Psychology*, 7th ed. Hoboken, NJ: Prentice Hall.

[B17] Ferreira A. Schwieter J. W. (2017). “Translation and cognition: An overview.” in *The Handbook of Translation and Cognition*, eds Schwieter J. W. Ferreira A. (New York, NY: Wiley-Blackwell), 1–17. 10.1002/9781119241485.ch1.

[B18] Gorozhanov A. I. Kosichenko E. F. Guseynova I. A. (2018). Teaching written translation online: theoretical model, software development, interim results. in *Proceedings of the The International Scientific and Practical Conference “Current Issues of Linguistics and Didactics: The Interdisciplinary Approach in Humanities and Social Sciences” (CILDIAH-April 23-28, 2018)* (Vol. 50), eds Cindori S. Larouk O. Malushko E. Y. Rebrin L. N. Shamne N. L. (Volgograd: SHS Web of Conferences), 1–6. 10.1051/shsconf/20185001062

[B19] Hakverdi M. Gücüm B. Korkmaz H. (2007). Factors influencing pre-service science teachers’ perception of computer self-efficacy. *Asia Pacific Forum Sci. Learn. Teach.* 8 1–14.

[B20] Haro-Soler M. M. (2017). Teaching practices and translation students’ self-efficacy: the teachers’ perceptions. *Curr. Trends Transl. Teach. Learn.* 4 198–228.

[B21] Haro-Soler M. M. (2019). Vicarious learning in the translation classroom: how can it influence students’ self-efficacy beliefs? *Engl. Stud. NBU* 5 92–113. 10.33919/esnbu.19.1.5

[B22] Haro-Soler M. M. Kiraly D. (2019). Exploring self-efficacy beliefs in symbiotic collaboration with students: an action research project. *Interpret. Transl. Train.* 13 255–270. 10.1080/1750399X.2019.1656405

[B23] Kalyanasundaram P. Madhavi C. (2020). Students’ perception on elearning with regard to online value added courses. *Int. J. Manag. (IJM)* 11 89–96.

[B24] Kelly D. (2008). Training the trainers: Towards a description of translator trainer competence and training needs analysis. *TTR Traduction Terminologie Rédaction* 21 99–125. 10.7202/029688ar 33270396

[B25] Khorrami-Arani O. (2001). Researching computer self-efficacy. *Int. Educ. J.* 2 17–25.

[B26] Kinzie M. B. Delcourt M. A. Powers S. M. (1994). Computer technologies: attitudes and self-efficacy across undergraduate disciplines. *Res. High. Educ.* 35 745–768. 10.1007/BF02497085

[B27] Linnenbrink E. A. Pintrich P. R. (2003). The role of self-efficacy beliefs in student engagement and learning in the classroom. *Read. Writ. Q.* 19 119–137. 10.1080/10573560308223

[B28] Mashhady H. Fatollahi M. Pourgalavi M. (2015). Self-efficacy and prediction of note-taking inclination among undergraduate translation students. *Theory Pract. Lang. Stud.* 5 2366–2372. 10.17507/tpls.0511.22

[B29] Monacelli C. (2009). *Self-Preservation in Simultaneous Interpreting.* Amsterdam: John Benjamins Publishing Company. 10.1075/btl.84

[B30] Núñez J. L. Bolaños-Medina A. (2018). Predictors of problem-solving in translation: Implications for translator training. *Interpret. Transl. Train.* 12 282–298. 10.1080/1750399X.2017.1359762

[B31] O’Brien S. Ehrensberger-Dow M. Connolly M. Hasler M. (2017). Irritating CAT tool features that matter to translators. *HERMES J. Lang. Commun. Bus.* 56 145–162. 10.7146/hjlcb.v0i56.97229

[B32] Obdrzalkova V. (2016). Translation as a decision-making process: An application of the model proposed by Jiri Levy to translation in to a non-mother tongue. *Mutatis Murandis Mutatis Mutandis, Rev. Latinoam. Traducción* 9 306–327.

[B33] Odacioglu M. C. Kokturk S. (2015). The effects of technology on translation students in academic translation teaching. *Proc. Soc. Behav. Sci.* 197 1085–1094. 10.1016/j.sbspro.2015.07.349

[B34] Özçelik H. Kurt A. A. (2007). Primary school teachers’ computer self efficacies: sample of Balkesir. *Ýlköğretim Online* 6 441–451.

[B35] Pietrzak P. (2020). Inside and outside the translation classroom. *Res. Lang.* 18 109–117. 10.18778/1731-7533.18.2.07

[B36] Pietrzak P. Kornacki M. (2021). *Using CAT Tools in Freelance Translation: Insights from a Case Study.* New York, NY: Routeledge. 10.4324/9781003125761

[B37] Risku H. Rogl R. Milosevic J. (2017). Translation practice in the field: current research processes. *Transl. Spaces* 6 3–26. 10.1075/ts.6.1.01ris 33486653

[B38] Rojo A. (2015). Translation meets cognitive science: the imprint of translation on cognitive processing. *Multil. J. Cross Cult. Interlang. Commun.* 34 721–746. 10.1515/multi-2014-0066

[B39] Rojo A. Ramos M. Valenzuela J. (2014). The emotional impact of translation: a heart rate study. *J. Pragmat.* 71 31–44. 10.1016/j.pragma.2014.07.006

[B40] Rojo-López A. M. Naranjo B. (2021). Translating in times of crisis: a study about the emotional effects of the Covid19 pandemic on the translation of evaluative language. *J. Pragmat.* 176 29–40. 10.1016/j.pragma.2021.01.018 PMC9759097 36568524

[B41] Saadeh D. Al-Karimi Q. (2009). “Blended e-learning approach at the University of Jordan.” in *The 4th International Conference on Information Technology (ICIT’09) Conference Proceedings Book*, eds Tamimi A. A. Al-Dahoud A. (Amman: Al-Zaytoonah University of Jordan), 1–6

[B42] Scherbaum C. A. Cohen-Charash Y. Kern M. J. (2006). Measuring general self-efficacy: a comparison of three measures using item response theory. *Educ. Psychol. Meas.* 66 1047–1063. 10.1177/0013164406288171

[B43] Scholz U. Doña B. G. Sud S. Schwarzer R. (2002). Is general self-efficacy a universal construct? *Eur. J. Psychol. Assess.* 18 242–251. 10.1027//1015-5759.18.3.242

[B44] Schunk D. H. Hanson A. R. (1985). Peer models: influence on children’s self-efficacy and achievement. *J. Educ. Psychol.* 77 313–322. 10.1037/0022-0663.77.3.313

[B45] Schwarzer R. Jerusalem M. (1995). “Generalized self-efficacy scale.” In *Measures in Health Psychology: A User’s Portfolio: Causal and Control Beliefs* eds Weinman J. Wright S. Johnston M. (Windsor, UK: NFER-Nelson Publishing Company), 35–37

[B46] Shih C. Y.-Y. (2015). Problem-solving and decision-making in translation revision: two case studies. *Across Lang. Cult.* 16 69–92. 10.1556/084.2015.16.1.4

[B47] Topkaya E. Z. (2010). Pre-service English language teachers’ perceptions of computer self-efficacy and general self-efficacy. *TOJET Turk. Online J. Educ. Technol.* 9 143–156.

[B48] Wood R. Atkins P. Tabernero C. (2000). Self-efficacy and strategy on complex tasks. *Appl. Psychol.* 49 430–446. 10.1111/1464-0597.00024

[B49] Yang X. Guo X. Yu S. (2016). Effects of cooperative translation on Chinese EFL student levels of interest and self-efficacy in specialized English translation. *Comput. Assist. Lang. Learn.* 29 477–493. 10.1080/09588221.2014.991794

[B50] Yang Y. Cao X. Huo X. (2021). The psychometric properties of translating self-efficacy belief perspectives from Chinese learners of translation. *Front. Psychol.*, 12:642566. 10.3389/fpsyg.2021.642566 33889115 PMC8055932

[B51] Yilmaz M. Gerçek C. Köseoğlu P. So H. (2006). An analysis of the self-efficacy beliefs about computers of biology teacher candidates in Hacettepe University. *Hacettepe Univ. J. Educ.* 30 278–287.

[B52] Zhang X. Ardasheva Y. (2019). Sources of college EFL learners’ self-efficacy in the English public speaking domain. *Engl. Specif. Purp.* 53 47–59. 10.1016/j.esp.2018.09.004

[B53] Zhu L. (2020). A critical review of the research on translation psychology: theoretical and methodological approaches. *Linguistica Antverpiensia. N. Ser. Themes Transl. Stud.* 19 53–79. 10.52034/lanstts.v19i0.559

